# Detection of fake-video uploaders on social media using Naive Bayesian model with social cues

**DOI:** 10.1038/s41598-021-95514-5

**Published:** 2021-08-09

**Authors:** Xiaojun Li, Shaochen Li, Jia Li, Junping Yao, Xvhao Xiao

**Affiliations:** 1 Xi’an Research Institute of High-Tech, Xi’an, 710025 China; 2 School of Business, East China University of Science and Technology, Shanghai, 200237 China

**Keywords:** Information technology, Software

## Abstract

With the rapid development of the Internet, the wide circulation of disinformation has considerably disrupted the search and recognition of information. Despite intensive research devoted to fake text detection, studies on fake short videos that inundate the Internet are rare. Fake videos, because of their quick transmission and broad reach, can increase misunderstanding, impact decision-making, and lead to irrevocable losses. Therefore, it is important to detect fake videos that mislead users on the Internet. Since it is difficult to detect fake videos directly, we probed the detection of fake video uploaders in this study with a vision to provide a basis for the detection of fake videos. Specifically, a dataset consisting of 450 uploaders of videos on diabetes and traditional Chinese medicine was constructed, five features of the fake video uploaders were proposed, and a Naive Bayesian model was built. Through experiments, the optimal feature combination was identified, and the proposed model reached a maximum accuracy of 70.7%.

## Introduction

The wide adoption of computers, smartphones, and other electric terminals, which increase the number of channels and the speed of information transmission, has boosted the development of the Internet. Take China as an example. As of June 2020, China had 940 million Internet users and an Internet penetration rate of 67.0%; mobile phone users reached 932 million, 99.2% of whom used mobile phones to go online; users of online videos (including short videos) totaled 888 million, accounting for 94.5% of all Internet users, among which 818 million were short video users, accounting for 87.0% of all Internet users^1^. These numbers provide a glimpse into the profound impact of the Internet on people’s daily lives and showcase the fact that short videos have already become an important channel for information acquisition.

The Internet, boasting large volumes of multimedia materials and quick information transmission, has become the major channel for multimedia information transmission. Benevenuto et al.^2^ suggest that video chatting, video mails and video blogs provide novel ways for interactions among Internet users, and many Internet service providers are displacing text-based services by video-based alternatives, such as video reviews, video advertisements and video replies. Though legitimate and friendly short videos are convenient to watch, illegitimate and fake videos are misleading. Pantazi et al.^3^ found that explicitly false statements, if considered true, would impact users’ judgments. In particular, false videos intended to mislead the audience will affect the audience’s judgment and their subsequent decision-making.

Misinformation, disregarding the scope and speed of transmission, brings only limited trouble to users, but if the misinformation is delivered in the form of videos, it reaches a wider audience at a higher speed. Thus, it is necessary to detect fake videos. Fake videos can be detected based on its content or source. However, most extant studies on fake video detection focus on its content^4,5^. Although the detection of fake reviewer has been investigated by many prior studies^6,7^, the detection of fake video uploaders is still lacking. Detection of fake video uploaders is the key step to identify the bad intention of fake videos. Some fake videos are misleading because the uploaders are ignorant. That does not mean the uploaders are intentionally misleading the public to get some benefits. However, if a user is identified as a fake video uploader, we can make sure that the videos he/she uploaded have bad intentions. To address the important research gap, we explores the possibility of detecting fake-video uploaders in this study.

This paper is comprised of five sections. Section 2 is a review of related works, Sect. 3 presents the Naive Bayesian model and the dataset of fake video uploaders used in this work, Sect. 4 discusses the experimental results and analysis, and Sect. 5 presents the conclusions.

## Related works

Since the results of literature review suggest that researches on detection of fake video uploaders is lacking, we decide to review the following two parts: (1) Fake video content detection; (2) Spammer detection in social media.

### Fake video content detection

The detection of fake video content has attracted many researchers in recent years. Extant studies on fake video content detection mainly focus on fake portrait video (e.g., generated by DeepFake), clickbait video or misleading video.

#### Detection of fake portrait video

The first stream of researches focus on the detection of fake portrait videos. Fake portrait video generation techniques are now a threat to the society with photorealistic deep fakes for political propaganda, celebrity imitation, forged evidences, and other identity related manipulations. Therefore, it is relevant to separate deep fake portrait videos from real videos. Ciftci et al.^4^ proposed a deep fake source detection via interpreting residuals with biological signals. They found that spatiotemporal patterns in biological signals can be conceived as a representative projection of residuals. Yu et al.^8^ proposed a novel method to detect the fake high definition for high efficient video coding videos based on prediction mode feature. Choi et al.^5^ proposed a novel fake video detection method by adopting predictive uncertainty in detection. They devised the certainty-based attention network which guides to focus certainty-key frames in detecting fake videos.

#### Detection of clickbait video

The second stream of researches focus on the detection of clickbait videos. Clickbait is a sensationalized headline that encourages you to click a link to an article, image, or video. Instead of presenting objective facts, clickbait headlines often appeal to your emotions and curiosity. However, the clickbait strategy typically values getting clicks over producing quality information. That means clickbait videos may waste the reader’s time with mediocre content. Therefore, many scholars have investigated how to detect clickbait videos. Varshney and Vishwakarma^9^ developed a Clickbait Video Detector (CVD) which considers three latent features as user profiling, video-content and human consensus. Shang et al.^10^ developed a novel content-agnostic scheme with the video comments. Different from existing solutions, this approach does not depend on the video content and its meta-data such as title or thumbnail. Jain et al.^11^ proposed a novel model to detect visual clickbaits on Instagram and Twitter. The proposed model consists of a stacking classifier framework composed of six base models and a meta-classifier.

#### Detection of misleading video

The third stream of researches focus on the detection of misleading videos, which is most relevant to the purpose of our study. However, the detection of misleading videos is a challenging task compared with the other two tasks mentioned above^12^. As a result, the detection of misleading videos was less investigated. Among the limited studies, Boididou et al.^13^ proposed a system that could classify multimedia Twitter posts into credible or misleading. The system leverages the credibility-oriented information extracted from the tweet content and the user identification. In another study, Papadopoulou et al.^14^ presented an approach based on supervised learning to classify misleading Web video. The proposed approach considers both video metadata (e.g., the video description, likes/dislikes, and uploader information) and the video comments.

### Fake content uploader detection in social media

The detection of fake content uploaders has also attracted many researchers in recent years. Extant studies on fake content uploader detection mainly focus on spammer detection and fake reviewer detection.

#### Detection of spammers

The first stream of researches focus on the detection of spammers. A spammer is a person or group that sends you email you don't want and didn't sign up for. The email a spammer sends is called spam. Spam is usually some kind of advertisement. Although spammers was first used to in the e-mail context, it is also soon applied to the social media context. Social spam is unwanted spam content appearing on social networking services, social bookmarking sites, and any website with user-generated content (comments, chat, etc.)

Many scholars have explored the detection of spammers in social media. For example, Hu et al.^15^ investigated how to collectively use network and content information to perform effective social spammer detection in microblogging. Yu et al.^16^ presented a novel semi-supervised social media spammer detection approach, making full use of the message content and user behavior as well as the social relation information. Miller et al.^17^ proposed an approach to detect twitter spammers using data stream clustering. Different from previous studies that approached spam detection as a classification problem, they viewed it as an anomaly detection problem. Park et al.^18^ proposed an approach to detect adaptive spammers that dynamically change their spamming strategies or behaviors and attempt to pose as legitimate users. This approach introduces a new method of moving target defense wherein the concept of differential immunity of different classifiers is employed to detect the spammers.

#### Detection of fake reviewers

The second stream of research focuses on the detection of fake reviewers. Fake reviewers try to deliberately mislead readers by giving undeserving positive opinions to some target entities in order to promote the entities and/or by giving false negative opinions to some other entities in order to damage their reputation. As opinions on the Web are increasingly used in practice by consumers, organizations, and businesses for their decision making, detecting fake reviewers is now a critical task for many e-commerce and rating platforms.

Many scholars have investigated the detection of individual fake reviewers. For example, Kumar et al.^6^ presented a fully unsupervised approach to detect anomalous behavior in online reviewers. They proposed a novel hierarchical approach in which they first derive distributions for key features that define reviewer behavior, and then combine these distributions into a finite mixture model. Zhang et al.^7^ proposed an improved label propagation algorithm with a propagation intensity and an automatic filtering mechanism to find candidate spammer groups based on the constructed reviewer relationship graph.

Many fake reviewers collaborate to cheat review readers in practice. Therefore, fake reviewer group detection has also be investigated in the literature. For example, Thahira and Sabitha^19^ proposed a framework to detect spammer groups using graph-based algorithms with five group spamming features and also proposed a new group spamming feature, group rating similarity based on the review rating score. Wang et al.^20^ proposed a graph-based review spammer group detection method. They introduced a top-down computing framework, namely GGSpam, to detect review spammer groups by exploiting the topological structure of the underlying reviewer graph which reveals the co-review collusiveness.

The above literature review suggests that both fake video content detection and fake reviewer detection have received a lot attention. However, the work on their combination (the detection of fake video uploaders) is still lacking in the literature. Considering the explosive growth of short videos on social media, the detection of false video uploaders has become an important practical issue. To address the research gap, we proposed a Naive Bayesian model that extracts features of fake video uploaders for detection.

## Naive Bayesian model and dataset of fake video uploaders

In this section, the extraction of features of fake video uploaders and the Naive Bayesian model are introduced, and the dataset used in the study is described.

### Feature extraction

As the extracted features have considerable impact on the accuracy of machine learning models, a set of effective features is necessary to detect fake video uploaders. In this work, features that can be directly collected are called observed features, such as user name, user signature, number of following accounts, number of followers, and number of likes, while the combinations of observed features are called high-dimensional features. All these features are extracted from video uploaders to differentiate legitimate users from fake video uploaders. The following five features were proposed and used in this study:

**1) Followers fans (FF)**, which measures the followed accounts and fans of the uploaders to be detected. FF is expressed as the ratio of fans of a user to the number of accounts the user follows:

1 $$ FF = \frac{{FVP_{{number_{{}} }} }}{{FVP_{attention} }} $$

where $FVP_{{number_{{}} }}$ is the number of fans of the user to be detected, and $FVP_{attention}$ is the number of accounts that the user to be detected follows. Setiawan et al.^21^ employed the ratio of fans of a user to the number of accounts that the user follows as a feature for the detection of spammers on Twitter. In this work, we consider this ratio an effective feature for the detection of spammers, and by analogy, the ratio of fans to the number of followed accounts of a user is used as an extraction feature.

**2) Average like (Al)**. People are more likely to believe what they think is correct. For example, eating leeks can reduce hyperglycemia. Although this is false, many people still believe it because it can satisfy people's expectations for good things. Compared with legitimate users, fake video uploaders are more likely to cater to people's expectations to mislead video watchers. Al refers to the average likes an uploader gets from the videos he/she uploads, expressed as:

2 $$ AL = \frac{{FVP_{likes} }}{{FVP_{views} }} $$

where $FVP_{likes}$ is the number of likes that the uploader obtains for the videos uploaded, and $FVP_{views}$ is the number of videos uploaded by the uploader.

**3) Search proportion (SP)**. A legitimate user, when seeing a video of interest, is very likely to click the like button, visit the main page of the uploader, and follow the uploader. Therefore, the number of fans should be less than the number of users who have watched the uploaded video. When the number of people who watch the uploaded video is much smaller than the number of fans, this is an abnormal phenomenon. A fake video uploader, however, directs the users to search his/her information and become fans. This can expand his/her influence and achieve the purpose of misleading more people. The SP is the ratio of the number of views to the number of fans:

3 $$ SP = \frac{{FVP_{reading} }}{{FVP_{{number_{{}} }} }} $$

where $FVP_{reading}$ is the number of users who have watched the uploaded video.

**4) Signature similarity (SS)**, which refers to the similarity between the fake-video uploader’s user name and signature. Syafii et al.^22^ employed similarity criteria and the entropy interval in experiments to detect spammers. Likewise, we introduced the similarity method to the detection of fake video uploaders. Legitimate users often do not pay much attention to their signatures, but fake-video uploaders will try their best to make their signature in line with their user name to convince other users of their authenticity. The cosine similarity between the user name and the signature is employed to calculate SS:

4 $$ SS = cosine\left( {FVP_{name} ,FVP_{signature} } \right) $$

where $FVP_{name}$ is the user name of the video uploader, and $FVP_{signature}$ stands for the signature of the uploader.

**5) Time difference (TD)** refers to the last time a video is uploaded. Mukerjee et al.^23^ detected spammers by account activeness, which was measured by the gap between the first time and the last time a review was posted and achieved sound results. In this work, we employed the interval between the last time the user uploads a video and the set time to detect fake-video uploaders because legitimate users release videos from time to time, while fake-video uploaders release a video only when necessary to mislead users. The equation for TD is:

5 $$ TD = FVP_{new} - T\left( a \right) $$

where $FVP_{new}$ is the time the last video is uploaded, and $T\left( a \right)$ is the time of data collection, which is a constant.

### Model

**Figure 1:**
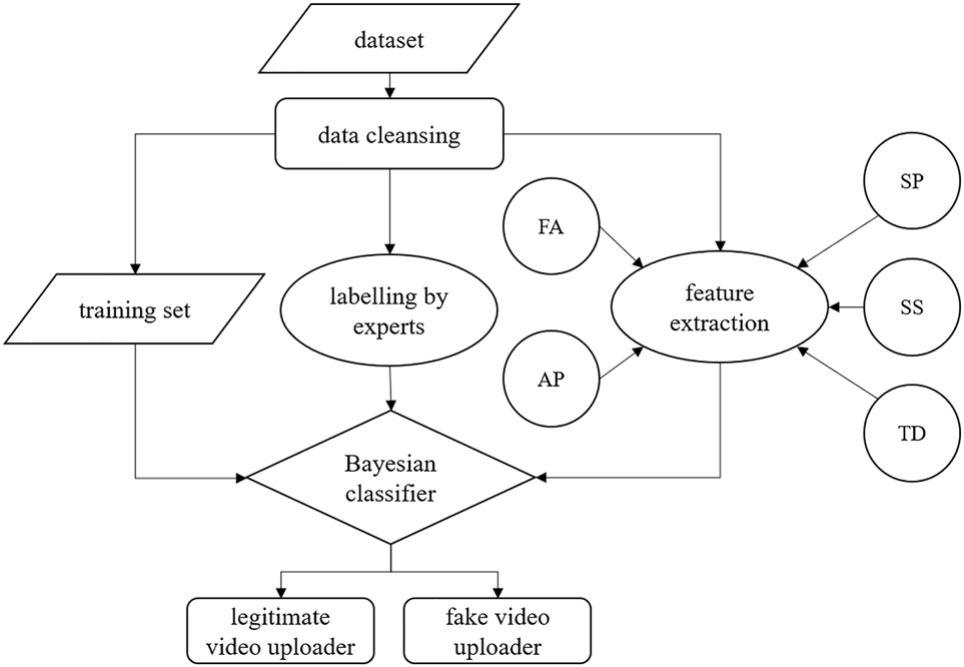
Workflow for detection of fake-video uploaders.

Figure 1 shows the workflow for the detection of fake video uploaders. The obtained data are first cleansed to remove anomalies and repetitive data; then, the following three steps are performed: (1) classification methods such as cross validation are employed to divide the cleansed dataset into a training set and a test set; (2) experts in corresponding research fields are invited to label the cleansed dataset; and (3) the feature extraction method detailed in Sect. 3.1 is used to extract features. Finally, the Bayesian classifier is employed to classify the video uploaders based on the three types of data obtained in the preceding three steps to judge whether the uploader is a fake-video uploader.

**Figure 2:**
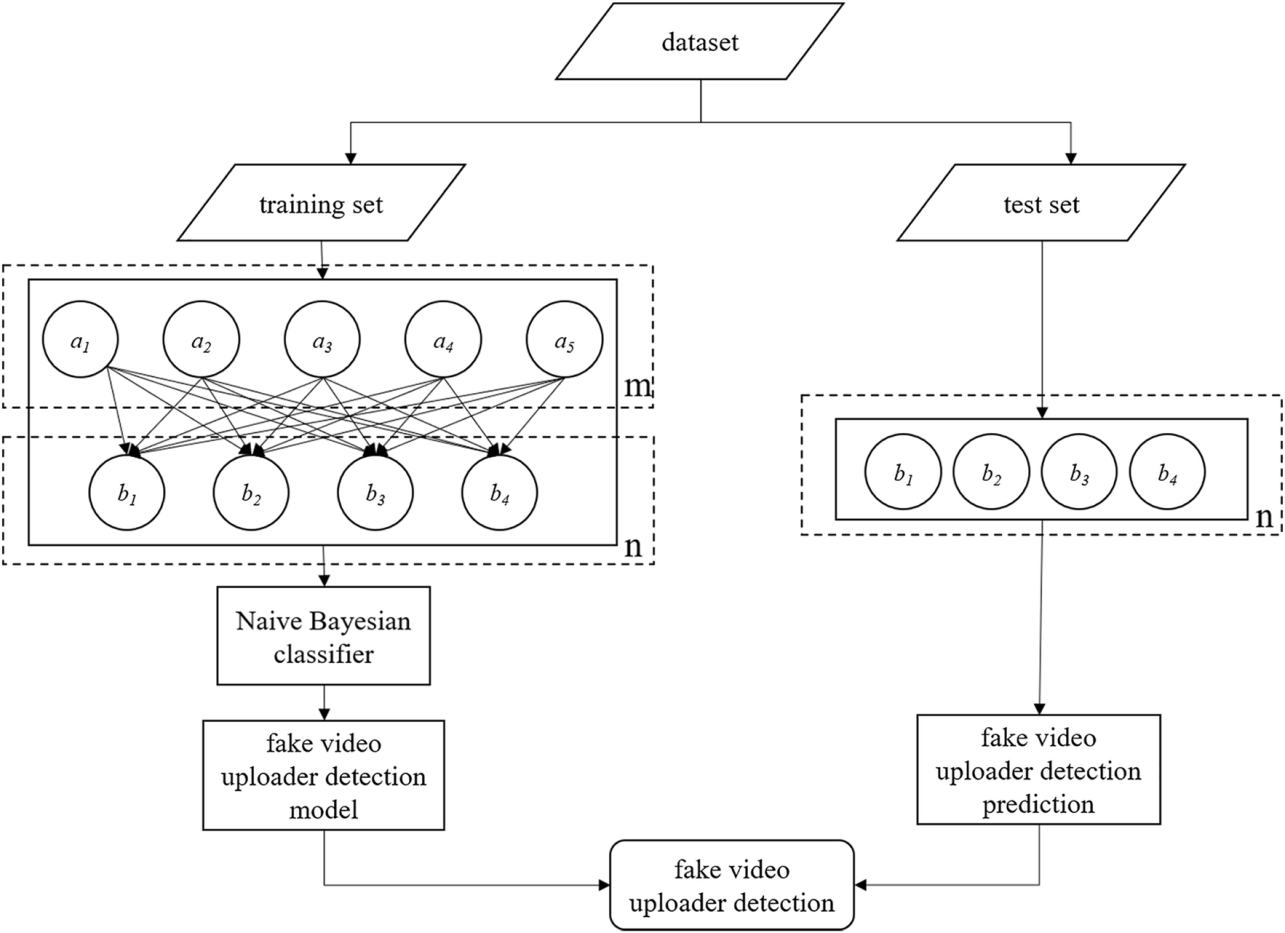
Fake video uploader detection model (features in dotted box m are attributes of false video publisher; features in dotted box n are fake-video uploader features).

In the modeling process in this work, the detection of fake-video uploaders was converted into a supervised classification problem, and the model simulated the differences between video uploaders. In the model, it was assumed that the fake- video uploaders to be tested were independent from the observed features, which is a premise of the Naive Bayesian model. Meanwhile, the observed features were processed into high-dimensional features, which were then compared with the labeled data by experts, to train the Bayesian classifier. Finally, the cross-validation method was employed to construct a test set and a training set to assess the model’s detection efficiency. The naive Bayesian model was employed here for the following three reasons. First, it is fast, has low complexity and requires no complicated procedures in the detection of the fake video uploaders. Second, the features used in this study are independent from each other, and the Bayesian classifier has good performance on independent features. Third, the sample size in this study is small. The Bayesian classifier, which does not require large quantities of samples, turns out a wise option. Figure 2 shows the fake video uploader detection model.

When using the Naive Bayesian model to detect fake-video uploaders, we assume that a dataset ${\rm X} = \left\{ {x_{1} ,x_{2} , \cdots ,x_{n - 1} ,x_{n} } \right\}$ is an unclassified set of video uploaders, where $x = \left\{ {a_{1} ,a_{2} , \cdots ,a_{n - 1} ,a_{n} } \right\}$ represents an unknown uploader, and $a$ is the attribute value of $x$. The uploader category set is ${\rm B} = \left\{ {y_{1} ,y_{2} } \right\}$, where $y_{1}$ represents a legitimate uploader, while $y_{2}$ stands for a fake-video uploader. For any video uploader $x$, the larger of $P\left( {y_{1} {|}x} \right)$ and $P\left( {y_{2} {|}x} \right)$ determines the category to which he/she belongs.

As per the Bayesian principle, we obtained:

6 $$ P\left( {y_{i} {|}x} \right) = \frac{{P\left( {x{|}y_{i} } \right)P\left( {y_{i} } \right)}}{P\left( x \right)} $$

In a Naive Bayesian model, it is assumed that all video uploaders are independent from the observed features, so $P\left( x \right)$ is a constant. Meanwhile, as the number of legitimate users and fake-video uploaders are known in a dataset, the value of $P\left( {y_{i} } \right)$ is known. Thus, when $P\left( {x{|}y_{i} } \right)$ is assigned the maximum value, $P\left( {y_{i} {|}x} \right)$ reaches the maximum; hence,

7 $$ \mathop {max}\limits_{0 < i \le 2} P\left( {x{|}y_{i} } \right) = \mathop {max}\limits_{0 < i \le 2} \mathop \prod \limits_{j = 1}^{n} P\left( {a_{j} {|}y_{i} } \right) $$

where $P\left( {a_{j} {|}y_{i} } \right)$ represents the probability that a video uploader attribute corresponds to a video uploader category.

As the Naive Bayesian model is highly dependent on the estimation of the conditional probability^24^, i.e., the prior probability has a large impact on the detection accuracy of the model, the prior knowledge matrix was employed to determine the hyperparameters in the prior distribution. We assume that the probability that a fixed attribute $a$ corresponds to the category in the category set $B$ conforms to a binomial distribution $B\left( {k,\theta } \right)$, where the prior distribution of $\theta$ is the Beta distribution $Be\left( {b,c} \right)$, and $b$ and $c$ are two hyperparameters. First, as per prior knowledge such as historical or empirical data, the multiple estimated values of $\theta$ (marked as $\theta_{1} ,\theta_{1} , \cdots ,\theta_{l}$), the prior mean $\overline{\theta }$, and prior variance $S_{\theta }^{2}$ are obtained:

8 $$ \overline{\theta } = \frac{1}{l}\mathop \sum \limits_{i = 1}^{l} \theta_{i} $$

9 $$ S_{\theta }^{2} = \frac{1}{l - 1}\mathop \sum \limits_{i = 1}^{l} \left( {\theta_{i} - \overline{\theta }} \right)^{2} $$

Let the prior mean and prior variance be the mean and variance of the Beta distribution $Be\left( {b,c} \right)$; then:

10 $$ \left\{ {\begin{array}{*{20}c} {\frac{b}{b + c} = \overline{\theta }} \\ {\frac{bc}{{\left( {b + c} \right)^{2} \left( {b + c + 1} \right)}} = S_{\theta }^{2} } \\ \end{array} } \right. $$

and we calculate estimates for the hyperparameters $b$ and $c$:

11 $$ \hat{b} = \overline{\theta }\left[ {\frac{{\left( {1 - \overline{\theta }} \right)\overline{\theta }}}{{S_{\theta }^{2} }} - 1} \right] $$

12 $$ \hat{c} = \left( {1 - \overline{\theta }} \right)\left[ {\frac{{\left( {1 - \overline{\theta }} \right)\overline{\theta }}}{{S_{\theta }^{2} }} - 1} \right] $$

The Beta distribution $Be\left( {b,c} \right)$ is assigned as the prior distribution of $\theta$, specified as follows:

We assume $x = \left( {x_{1} ,x_{2} , \cdots ,x_{n} } \right)$ is a sample from the density function $f\left( {x{|}\theta } \right)$, where $\theta = \left( {\theta_{1} ,\theta_{2} , \cdots ,\theta_{n} } \right)$ is a $p$-dimensional parameter vector.

Thus, the logarithm of the likelihood function of the sample is:

13 $$ L = ln\left[ {L\left( {x{|}\theta } \right)} \right] = ln\left[ {\mathop \prod \limits_{i = 1}^{n} f\left( {x_{i} {|}\theta } \right)} \right] = \mathop \sum \limits_{i = 1}^{n} lnf\left( {x_{i} {|}\theta } \right) $$

The Fisher information matrix is:

14 $$ I\left( \theta \right) = E\left( {\frac{{ - \partial^{2} L}}{{\partial \theta_{i} \partial \theta_{j} }}} \right)i,j = 1,2, \cdots ,p. $$

Then, the prior density function of $\theta$ is:

15 $$ \pi \left( \theta \right) = \left[ {detI\left( \theta \right)} \right]^{\frac{1}{2}} $$

Meanwhile, as $X$ conforms to a binomial distribution $B\left( {k,\theta } \right)$, i.e.,

16 $$ P\left( {X = x} \right) = C_{k}^{x} \theta^{x} \left( {1 - \theta } \right)^{k - x} ,x = 0,1,2 \cdots ,k. $$

we find that the distribution of $\theta$ is a Beta distribution.

Then, we can solve $P\left( {a_{j} {|}y_{i} } \right)$ and thus determine the category to which the unknown video uploader $x$ is most likely to belong.

### Dataset

The data in this study was collected from a famous video websites in China Bilibili (https://www.bilibili.com/). We collected the information about all video-uploaders who uploaded more than 3 videos about diabetes or traditional Chinese medicine (TCM). The meta-data for each video include user name, signature, the number of following accounts, the number of fans, the number of likes, play counts and the number of views. Meanwhile, two experts in the medical field were invited to label the collected video uploaders. The medical experts judged the authenticity of videos posted by video uploaders. The number of legitimate video uploaders and fake-video uploaders was balanced without reducing the validity of the research results. We randomly selected 225 fake-video uploaders and 225 legitimate uploaders to constitute the research data. tenfold cross-validation was used to split the training data and test data. That is, the 450 samples are randomly and equally divided into 10 groups. One groups is randomly selected as the test data, and the remaining 9 group is used as the training data. The averaged performance on 10 test data was used as the final performance. All experimental protocols were approved by the Ethics Committee in the School of Business, East China University of Science and Technology. All methods were carried out in accordance with relevant guidelines and regulations. Informed consent was obtained from all subjects or if subjects are under 18, from a parent and/or legal guardian. Table 1 lists the attributes and corresponding definitions. Table 2 describes the statistical values of related attributes.

**Table 1:** Attributes of video uploaders and corresponding definitions.

Attribute	Definition
name	User name of the video uploader
signature	Signature of the video uploader
attention	The number of accounts the uploader follows
number_of_fans	Fans the uploader has
likes	The sum of likes the uploader gets from the uploaded videos
views	The play count of all videos the uploader uploads
reading	Views the uploader gets
contributions	Number of works uploaded by the video uploader
channels	Number of channels the uploader has
videos	The sum of videos the uploader has uploaded
audios	The sum of audio works the uploader has uploaded
columns_count	The number of columns the uploader uploads
albums_count	The number of albums the uploader uploads
new_upload_time	The time the last video was released
is_FVP	Fake-video uploader labeled by the expert

**Table 2:** Statistics of related attributes.

Attribute	Average	Standard deviation
attention	59.51	149.64
number_of_fans	37,700.51	267,983.4
likes	131,059.70	978,923.04
views	5,256,729.45	56,734,091.54
reading	20,082.08	224,453.71
contributions	268.98	1052.97
channels	0.79	1.99
videos	240.72	1000.71
audios	2.97	50.04
columns_count	9.53	6342
albums_count	15.02	67.92

### Evaluation standards

When an untrained classifier is used, it is necessary to evaluate its performance in classifying unknown samples, and the evaluation indicators often include accuracy, precision, and recall^25^. In this work, accuracy, precision, recall, and F-score were employed as the performance indicators. Table 3 shows the confusion matrix of the research results.

**Table 3:** Confusion matrix in this study.

Category ${\varvec{C}}_{{\varvec{i}}}$	Actual result	
Prediction result	Yes	No
Yes	$TP$	$FP$
No	$FN$	$TN$

*TP* true positives, *FP* false positives, *FN* false negatives, *TN* true negatives.

The “Accuracy” describes the percentage of correct classification of legitimate and fake-video uploaders by the classifier, which is expressed as:

17 $$ Accuracy = \frac{TP + TN}{{TP + FP + FN + TN}} $$

The “Precision” describes the percentage of true fake-video uploaders in all the fake-video uploaders detected by the classifier, which is expressed as:

18 $$ Precision = \frac{TP}{{TP + FP}} $$

The “Recall” describes the probability of detecting the actual fake-video uploaders, which is expressed as:

19 $$ Recall = \frac{TP}{{TP + FN}} $$

The F-score is a comprehensive measure of the classifier that combines the accuracy and recall, which is expressed as:

20 $$ F - score = \frac{2 \times Accuracy \times Recall}{{Accuracy + Recall}} $$

## Experimental results and analysis

This section presents the experimental results of using the Naive Bayesian classifier to classify the extracted features. The experiments were performed on Python 3.6.12.

### Experimental results

The features of the dataset were extracted as per the methods detailed in Sect. 3.1., and the extracted features were classified. Table 4 presents the relevant features and classification results.

**Table 4:** Feature extraction of fake-video uploaders and classification results.

Feature combination		Classification results			
		Accuracy	Precision	Recall	F-score
1	FF + AL	54.1	63.1	50.9	56.4
2	FF + SP	57.2	37.4	59.0	45.8
3	FF + TD	57.2	37.4	59.0	45.8
4	AL + SP	55.9	50.9	54.5	52.6
5	AL + TD	55.9	50.9	54.5	52.6
6	SP + TD	52.2	58.4	50.9	54.4
7	SS + TD	69.8	88.7	62.7	73.5
8	FF + SS	69.8	88.7	62.7	73.5
9	AL + SS	69.8	88.7	62.7	73.5
10	SP + SS	69.8	88.7	62.7	73.5
11	FF + AL + SP	57.6	63.0	55.1	58.8
12	FF + AL + TD	57.4	56.6	55.7	56.1
13	FF + SP + TD	57.2	37.4	59.0	45.8
14	FF + AL + SS	69.8	88.7	62.7	73.5
15	FF + SP + SS	69.8	88.7	62.7	73.5
16	FF + SS + TD	69.8	88.7	62.7	73.5
17	AL + SP + SS	69.8	88.7	62.7	73.5
18	AL + SS + TD	69.8	88.7	62.7	73.5
19	SP + SS + TD	69.8	88.7	62.7	73.5
20	AL + SP + SS + TD	69.8	88.7	62.7	73.5
21	FF + SP + SS + TD	69.1	87.4	62.3	72.8
22	FF + AL + SS + TD	70.7	82.5	64.6	72.5
23	FF + AL + SP + SS	70.0	88.3	63.0	73.5
24	FF + AL + SP + TD	57.6	56.6	56.0	56.3
25	FF + AL + SP + SS + TD	70.0	88.3	63.0	73.5

**Figure 3:**
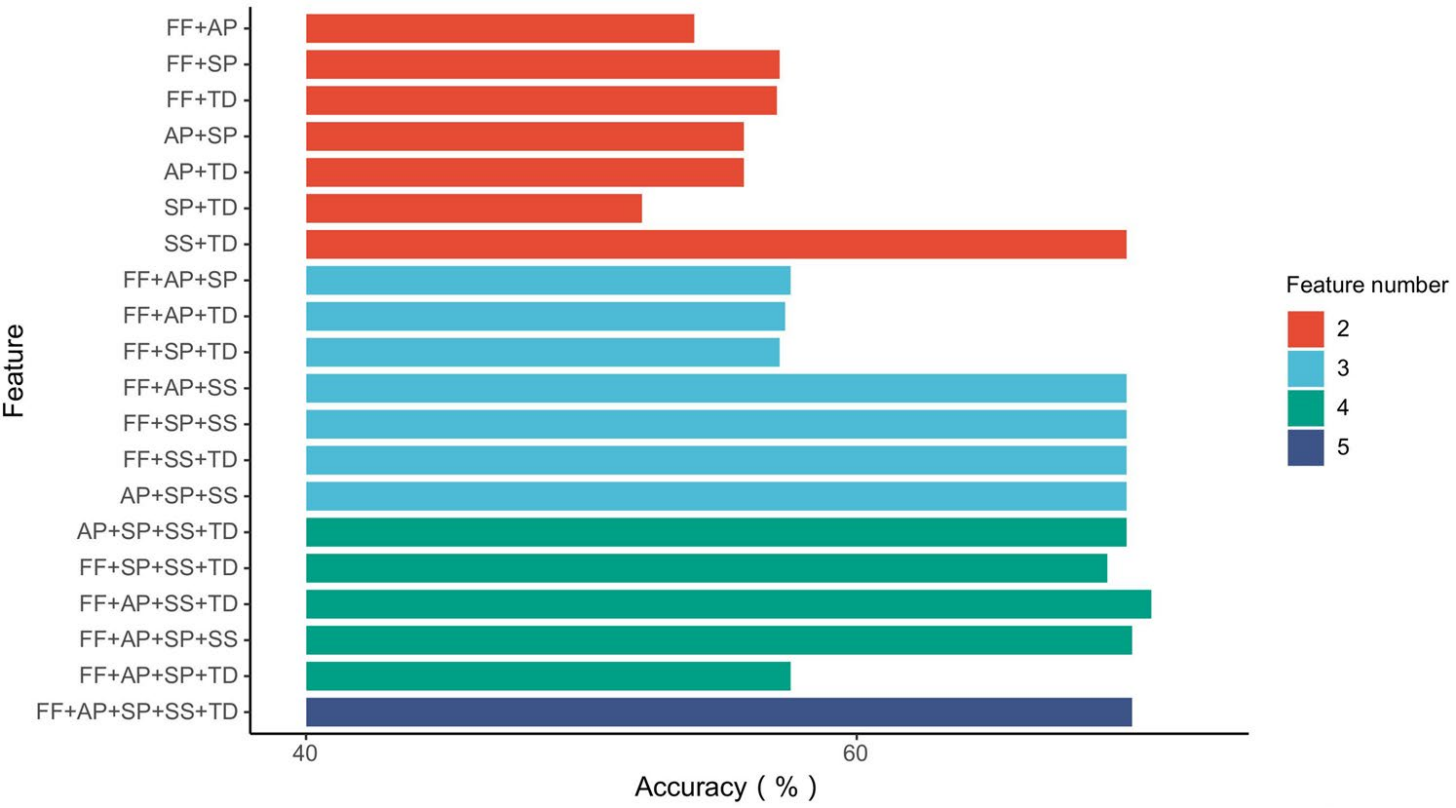
Accuracy of feature combinations by the Naive Bayesian model.

**Figure 4:**
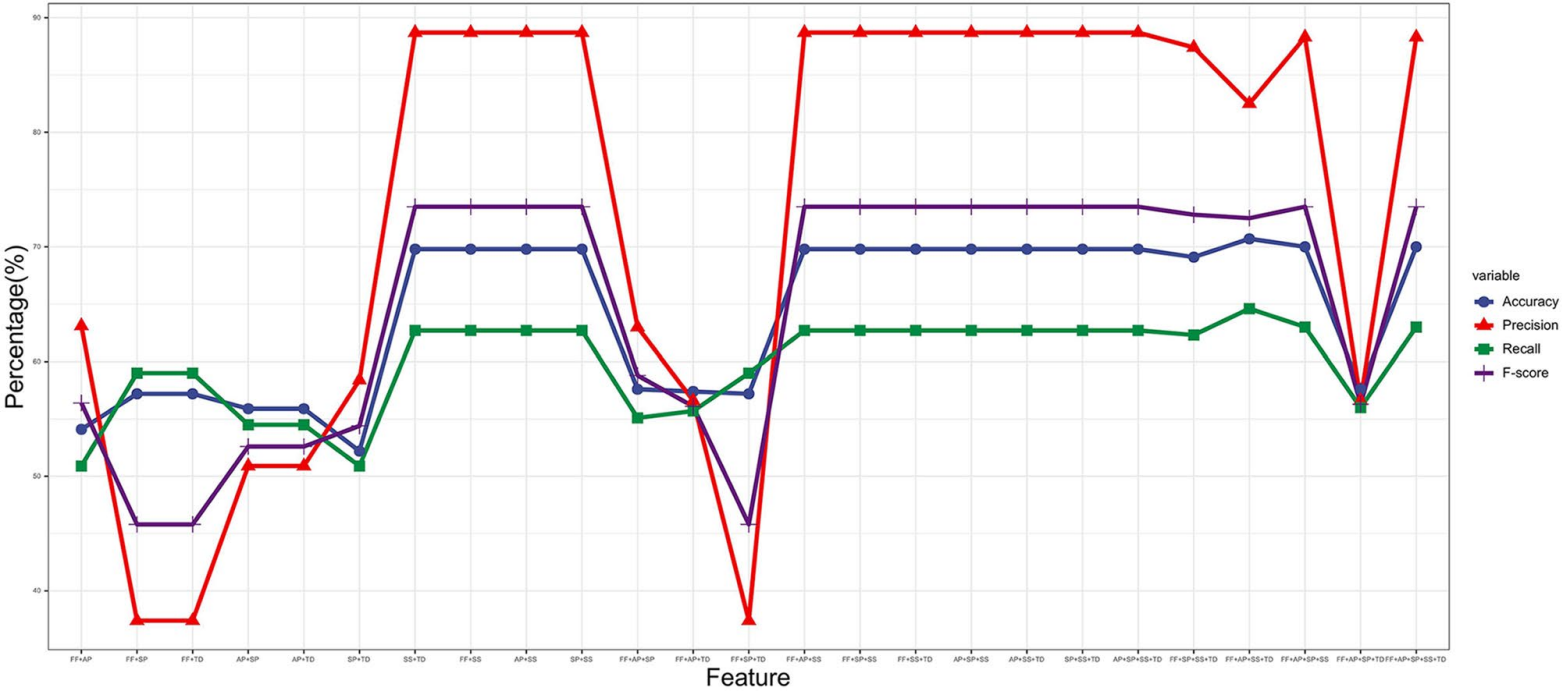
Classification results of the Naive Bayesian model based on different feature combinations.

Figures 3 and 4 present the classification results for different feature combinations.

To test the validity of the proposed approach (i.e., Naive Bayesian model), we also compare it with other popular baselines. The baselines chosen in this study include decision tree, random forest and Support Vector Machine (SVM). The comparison of proposed approach and other baselines are shown in Table 5. To simplify the result, we only report the best performance for each model in Table 5.

**Table 5:** The comparison of proposed approach (Naive Bayesian model) and other basslines.

Model	Accuracy	Precision	Recall	F-score
Decision Tree	0.62	0.57	0.65	0.60
Random Forest	0.69	0.74	**0.68**	0.71
SVM	0.47	0.73	0.52	0.49
**Naive Bayesian**	**0.71**	**0.83**	0.65	**0.73**

### Result analysis

In this work, the Naive Bayesian model was employed to classify the dataset. Figure 3 shows the accuracy for all feature combinations. As seen in the figures, combinations that include the SS feature (signature similarity) typically lead to a good classification result, which indicates that SS plays an important role in detecting fake-video uploaders by the Naive Bayesian model. SS reflects the similarity between the user name and the signature of the video uploader, which means that a fake video uploader is more likely to create a signature similar to his/her user name to deceive legitimate users.

Figure 4 presents the detection results of different feature combinations for the accuracy, precision, recall and F-score indicators. As the Figure shows, the feature combination used has a significant impact on the accuracy of the model (the accuracy ranges from 52.2% to 70.7%), where the SP-TD combination (No. 6 in Table 4) leads to an accuracy of merely 52.2%, while the FF-AL-SS-TD (No. 22 in Table 4) combination leads to the maximum accuracy of 70.7%. Meanwhile, comparing the results for combinations 1, 11 and 22 in Table 4, we find that as the number of features increases in a combination, the accuracy, recall and F-score all increase, which indicates a positive correlation between the number of features and the model’s performance.

**Figure 5:**
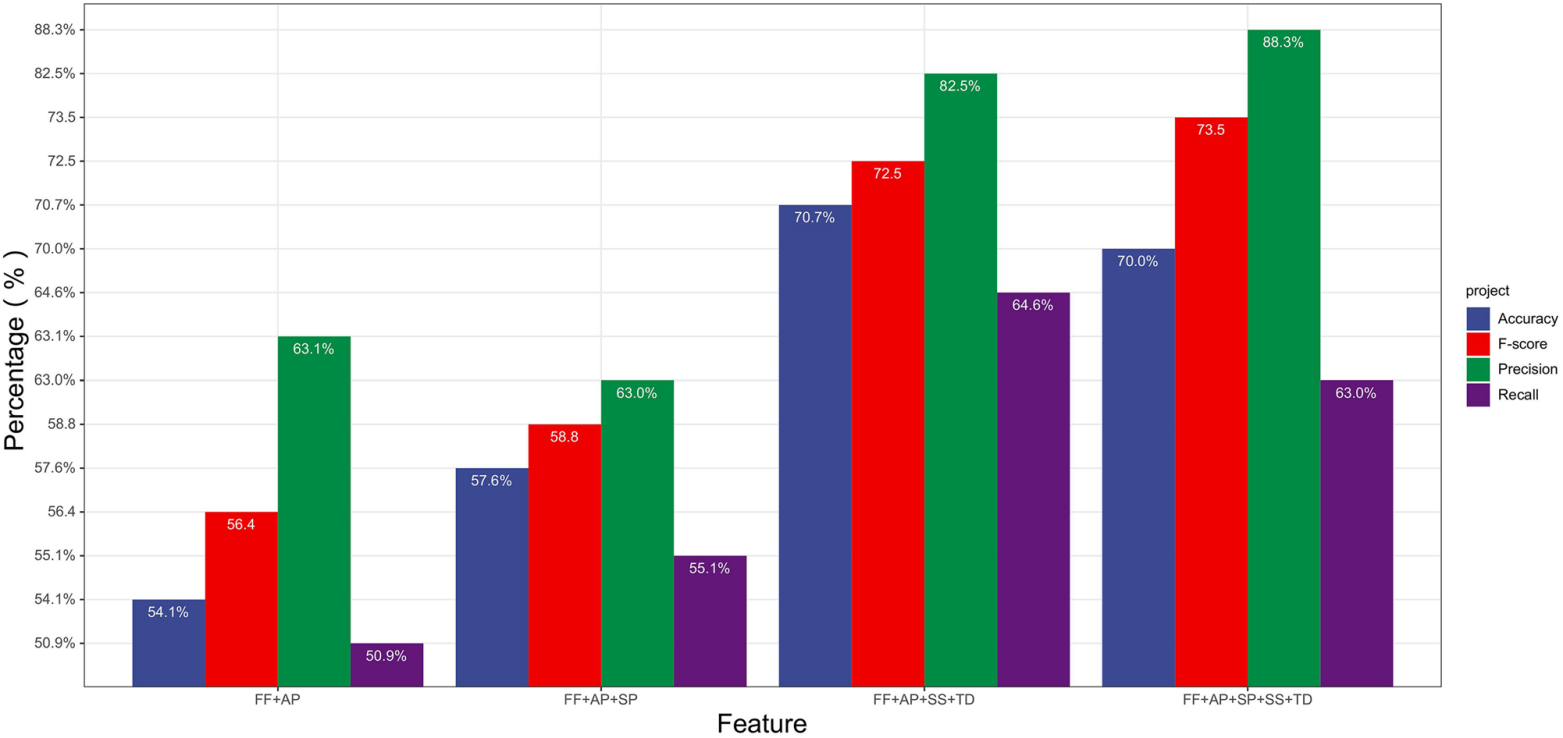
Comparison of classification results by the Naive Bayesian model based on different feature combinations.

Figure 5 compares the experimental results of the model on four different feature combinations based on the control variate method. This implies a positive correlation between the number of features and the model’s performance, but the correlation is not absolute. For instance, by comparing Combination 1 with Combination 11, we found that the introduction of the SP feature increased the accuracy, recall and F-score; although the accuracy declined slightly, this evidences the positive role of SP in improving the model’s performance. However, by comparing combinations 22 and 25, we found that the introduction of SP resulted in overfitting and hence a drop in the accuracy and recall. By probing further, we could see that though drawing fans through searching is a way for video uploaders to increase their fan count, it is also a channel for legitimate users to increase followers.

Table 5 compares the proposed approach and other baselines. As shown in Table 5, Naive Bayesian model outperforms all the baselines in accuracy, precision and F-score. One exception is that random forest (0.68) outperforms the Naive Bayesian model (0.65) in recall. Therefore, we can conclude that the proposed approach Naive Bayesian model in appropriate to solve the research question in this study.

## Conclusions

In this work, the detection of fake-video uploaders was discussed in detail. Specifically, a dataset consisting of 450 video uploaders and feature combinations was constructed, and experts were invited to label the uploaders; then, a Naive Bayesian model for the detection of fake video uploaders was built, and five detection features were proposed. Experiments were performed on different feature combinations, and the Naive Bayesian model reached a maximum detection accuracy of 70.7%.

Future research efforts can be made in the following two aspects. First, the Naive Bayesian model proposed in this study can be optimized to improve its detection accuracy. For instance, it is feasible to assign weights to the detection features as per their impact on the results and calculate the weights of different feature combinations. Second, the fake-video uploader detection results achieved in this study can be used as a basis to explore more detection methods and construct new detection models.

## References

[1] CNNIC. *The 46th Statistical Report on Internet Development in China* (2020).

[2] Benevenuto, F. *et al.* In *Proceedings of the 4th International Workshop on Adversarial Information Retrieval on the Web.* 45–52 (Association for Computing Machinery).

[3] Pantazi M, Kissine M, Klein O (2018). The power of the truth bias: False information affects memory and judgment even in the absence of distraction. Soc. Cogn..

[4] Ciftci, U. A., Demir, I. & Yin, L. In *2020 IEEE International Joint Conference on Biometrics (IJCB).* 1–10 (IEEE Press).

[5] Choi, D. H., Lee, H. J., Lee, S., Kim, J. U. & Ro, Y. M. In *2020 IEEE International Conference on Image Processing (ICIP).* 823–827 (IEEE Press).

[6] Kumar N, Venugopal D, Qiu L, Kumar S (2019). Detecting anomalous online reviewers: An unsupervised approach using mixture models. J. Manag. Inf. Syst..

[7] Zhang F, Hao X, Chao J, Yuan S (2020). Label propagation-based approach for detecting review spammer groups on e-commerce websites. Knowledge Based Systems.

[8] Yu Y, Yao H, Ni R, Zhao Y (2020). Detection of fake high definition for HEVC videos based on prediction mode feature. Signal Processing.

[9] Varshney, D. & Vishwakarma, D. K. A unified approach for detection of Clickbait videos on YouTube using cognitive evidences. *Applied Intelligence*, 1–22 (2021). 10.1007/s10489-020-02057-9 PMC7778503 34764575

[10] Shang L, Zhang DY, Wang M, Lai S, Wang D (2019). Towards reliable online clickbait video detection: A content-agnostic approach. Knowledge-Based Systems.

[11] Jain, M., Mowar, P., Goel, R. & Vishwakarma, D. K. In *2021 55th Annual Conference on Information Sciences and Systems (CISS).* 1–6 (IEEE Press).

[12] Papadopoulou O, Zampoglou M, Papadopoulos S, Kompatsiaris I (2019). A corpus of debunked and verified user-generated videos. Online Inf. Rev..

[13] Boididou C (2018). Detection and visualization of misleading content on Twitter. Int. J. Multimed. Inf. Retriev..

[14] Papadopoulou, O., Zampoglou, M., Papadopoulos, S. & Kompatsiaris, Y. In *Proceedings of the 2nd International Workshop on Multimedia Forensics and Security.* 6–10.

[15] Hu, X., Tang, J., Zhang, Y. & Liu, H. In *Twenty-Third International Joint Conference on Artificial Intelligence.* 2633–2639.

[16] Yu D, Chen N, Jiang F, Fu B, Qin A (2017). Constrained NMF-based semi-supervised learning for social media spammer detection. Knowl. Based Syst..

[17] Miller Z, Dickinson B, Deitrick W, Hu W, Wang AH (2014). Twitter spammer detection using data stream clustering. Inf. Sci..

[18] Park JH, Rathore S, Moon D, Park JH (2018). MTD-Spamguard: A moving target defense-based spammer detection system in social network. Soft. Comput..

[19] Thahira, A. & Sabitha, S. In *2021 2nd International Conference on Computation, Automation and Knowledge Management (ICCAKM).* 145–150 (IEEE Press).

[20] Wang Z, Gu S, Zhao X, Xu X (2018). Graph-based review spammer group detection. Knowl. Inf. Syst..

[21] Setiawan, E. B., Widyantoro, D. H. & Surendro, K. In *2018 6th International Conference on Information and Communication Technology (ICoICT).* 259–263 (IEEE Press).

[22] Syafii I, Setyanto A, Raharjo S (2018). Deteksi Bot Spammer Pada Twitter Menggunakan Smith Waterman Similarity Dan Time Interval Entropy. Jurnal RESTI (Rekayasa Sistem dan Teknologi Informasi).

[23] Mukherjee, A. *et al.* In *Proceedings of the 19th ACM SIGKDD international conference on Knowledge discovery and data mining.* 632–640.

[24] He Y-L, Wang R, Kwong S, Wang X-Z (2014). Bayesian classifiers based on probability density estimation and their applications to simultaneous fault diagnosis. Inf. Sci..

[25] Seref B, Bostanci E (2019). Performance of Naïve and complement Naïve Bayes algorithms based on accuracy, precision and recall performance evaluation criterions. Int. J. Comput..

